# The Common Missed Handwashing Instances and Areas after 15 Years of Hand-Hygiene Education

**DOI:** 10.1155/2019/5928924

**Published:** 2019-08-08

**Authors:** J. S. W. Wong, J. K. F. Lee

**Affiliations:** ^1^School of Nursing, Tung Wah College, 16/F 31 Wylie Road, Homantin, Kowloon, Hong Kong; ^2^AICU, Pamela Youde Nethersole Eastern Hospital, 3 Lok Man Road, Chai Wan, Hong Kong

## Abstract

The outbreak of severe acute respiratory syndrome (SARS) claimed the lives of 286 Hong Kong people in 2003. Since then, the Hong Kong government has been promoting the benefits of proper hand hygiene. There are few studies that explore the general quality of handwashing and the hand-hygiene practices of the public of Hong Kong; given this, the aim of this study is to explore this neglected topic. This study is a quantitative study that was conducted in January 2018. The results show that the majority of participants only wash their hands after using the toilet (87%) or handling vomitus or faecal matter (91%). The mean duration of handwashing was 36.54 seconds (SD = 18.57). The areas of the hand most neglected during handwashing were the fingertips (48.1%), medial area (30.5%), and back of the hand (28%). A multiple logistic regression shows that participants who have reached third-level education or higher often tend to be more hand hygienic than those who have not reached third-level education (*p* ≤ 0.001, *B* = 1.003). Thus, participants aged 30 and above tend to neglect 5 more areas of the hand than those aged below 30 (*p*=0.001, *B* = 4.933).

## 1. Introduction

In 2003, Hong Kong was profoundly affected by an unknown communicable disease entitled “severe acute respiratory syndrome” (SARS). The outbreak originated in a hospital and then spread to the wider community. The disease took the lives of 286 Hong Kong citizens and 774 people worldwide. In total, there were 8,096 known cases [1]. Given the seriousness of this outbreak, the promotion of the World Health Organization (WHO) guidelines and implementation of the “SAVE LIVES: Clean Your Hands” campaign became imperative. Furthermore, in 2004, the Hong Kong government established the Centre for Health Protection (CHP), a similar entity as the US's Centers for Disease Control and Prevention (CDC). The CHP aims to effectively prevent and control diseases in Hong Kong, in collaboration with both national and international stakeholders. Since the beginning of CHP, the agency has devised numerous guidelines and publications for health workers and the general public in order to prevent the spread of communicable diseases and promote healthy living. Following the SARS outbreak in Hong Kong, proper hand hygiene has been widely promoted in multiple contexts, focusing on the instances when hands should be washed as well as the duration and technique of handwashing. The worldwide public health agencies have asserted that proper hand hygiene can control the spread of communicable diseases from the common cold to the life-threatening severe acute respiratory syndrome, as well as fight against the rise in antibiotic resistance. Systematic reviews also show that insufficient washing of hands increases food-borne illness outbreaks and diarrheal diseases [2, 3]. However, there are few studies that evaluate the compliance of Hong Kong people in this area or the effectiveness of their handwashing after 15 years of health education on this topic. Thus, these are the main topics of interest for this study.

## 2. Literature Review

After the SARS outbreak and the development of hand-hygiene guidelines, researchers used various tools to evaluate the effectiveness of handwashing and alcohol-based hand sanitizers. The majority of handwashing and hygiene studies typically focused on healthcare providers and students, while few studies targeted the general public. The common areas of focus for these studies included the length of time spent handwashing, missed areas, common situations where handwashing was warranted, compliance with guidelines, and the relationship between demographic data and hand cleanliness.

For instance, in the US, Monk-Tuner et al. reported that only 10% of their 313 participants washed their hands for 15 seconds or longer. Drankiewicz and Dundes observed in their study that only 2% of participating university students washed their hands for 10 seconds or more [4]. Shanks and Peteroy-Kelly found that the average time university students spent washing their hands was a mere 4.87 seconds [5]. A observational study conducted by Michigan State University researchers also revealed that, among 3,749 people, the average washing times for men and women are 6.27 and 7.07 seconds, respectively, even though the CDC has been advocating the proper method for handwashing since 2012 [6].

In Turkey, Ergin et al. reported that 27% of 303 university students washed their hands just 4 times per day. They found that the main reason for students not washing their hands on a more regular basis was due to the belief that there was no need to do so. They also found that female participants scored significantly higher in knowledge, skills, and practices of hand hygiene [7]. In Greece, Mentziou et al. found that handrails and desks were the most frequently touched objects in universities, and the majority of university students performed handwashing after using the toilet and on returning home from university [8]. In Kenya, Curtis et al. also found that handwashing after using the toilet was the most common instance of hand hygiene [9, 10].

Also, previous studies have identified that gender, age, employment status, and educational level are the factors that affect an individual's hand-hygiene practices. In the US, Anderson et al., Berry et al., Edwards et al., and Vanyolos et al. found that female participants washed their hands significantly more than male participants; in Korea, Park et al. also found this to be true [11–14]. In the US, Duggan et al. found that there was a reciprocal relationship between professional education and handwashing compliance [15]. Burnett found that an increase in age was linked to improved perceptions of hand-hygiene practices [16]. Lau et al. found that factors such as age, employment status, and perceived local outbreaks of SARS were related to adopting good hand-hygiene practices for the protection of oneself and others [17]. In contrast, Pan et al. found that the number of areas of the hands that were neglected during handwashing had no correlation with the demographic data (gender, age, or profession) of participants; they also found no significant relationship between the duration of handwashing and the number of missed areas [18]. Some studies have revealed that exposure to proper hand-hygiene practices through mass media and the availability of handwashing facilities affect general handwashing practice [9, 10, 13].

Regarding the effectiveness of handwashing, Szilágyi et al. and Vanyolos et al. used fluorescent hand gel with ultraviolet (UV) light to assess the nursing and medical students' quality of handwashing [12, 19]. The results showed that women wash their hands better than men, with nurses displaying the best handwashing practices. When comparing different age groups, participants aged 40–49 performed the best overall. The most common missed areas were the fingernails and wrists [19]. A similar procedure using visual assessment was carried out by Kampf et al., and the results showed that the palms and fingertips were usually quite thoroughly cleaned [20]. In another study, Kampf et al. used gel containing UV-sensitive dye [21]. Participants were asked to apply this gel to their hands and then wash them under running water. A graphical assessment technique was then used to assess the missed areas by evaluating the absence of the UV dye. The tools used in this study make reference to the above-mentioned literature.

In Hong Kong, the CHP advocates proper handwashing practice to the public aligned with the CDC in the United States, the National Health Service (NHS) in the United Kingdom, the Public Health Agency of Canada, and the Global Handwashing Partnership which includes washing one's hands with water and soap before and after at least eight specific situations; the process comprises six steps over seven areas of both hands for no less than 20 seconds [22]. In 2014, the CHP conducted a hand-hygiene survey. The results revealed that although Hong Kong people had a good understanding of hand hygiene, 6% of them reported that they did not wash their hands after using the toilet, and less than a third washed their hands after touching public equipment or installations. Recently, Lee et al. developed an observational checklist in order to assess foreign domestic workers' handwashing practices in Hong Kong [23]. On average, they correctly performed 5 out of the necessary 13 handwashing steps. None of the participants rubbed their hands for 20 seconds or more, and none of the participants rubbed all areas of their hands. Moreover, a study was also conducted in a local hospital in relation to the compliance of healthcare professionals with the WHO's “My 5 Moments for Hand Hygiene” guidelines. The frequency of hand-hygiene practices was measured, but the quality of the practices was not evaluated. Of the 13,694 situations in which hand hygiene should have been practiced in a unit with 21 healthcare professionals, including nurses, physiotherapists, and healthcare assistants, the compliance rate was 35.1%. Lower rates of compliance were noted from 12:00 p.m. to 14:00 p.m. (21.3%, 95% CI: 17.2–25.3), as well as among nurses who shared their badges with others (23.7%, 95% CI: 17.8–29.6) [24]. Among the above-mentioned local studies, none of them was able to unveil the overall effectiveness of handwashing, and a comprehensive and in-depth exploration of hand-hygiene practices among Hong Kong people is neglected [25]. Therefore, this study was designed to fill this gap, and the following research questions were developed:

1. What are the most common instances of handwashing among Hong Kong adults?
2. How long do Hong Kong adults spend washing their hands?
3. What areas are most commonly missed in handwashing?
4. How many of the participants have received proper hand-hygiene information, and what are the common information resources?
5. What are the differences between independent variables, common missed areas, and handwashing instances?
6. What is the relationship between independent variables, common missed areas, and handwashing instances?

## 3. Methods

### 3.1. Methods

This study used a cross-sectional survey with convenience sampling, and behavioural observations were conducted from January to March in 2018. Before conducting this study, ethical approval was obtained from the research ethics committee of a local higher-education institute in Hong Kong. The ethical committee reference number is NUR/SRC/20171220/016.

### 3.2. Venue and Participants

Given that the target population was Hong Kong adults and that public handwashing facilities were required, this study was conducted in several public barbecue sites in three different territories of Hong Kong (i.e., Hong Kong Island, Kowloon, and the New Territories) in order to broaden the scope of this study. Barbeque sites were chosen as areas to recruit participants because spacious handwashing facilities with touchless faucets and sinks were provided in an outdoor washing area. The participants were the visitors to the barbecue sites whose age ≥18, mentally sound Hong Kong residents, and those who were able to communicate with Cantonese as Cantonese is the mother tongue of Hong Kong people. Participants who had experience working in healthcare settings and those with artificial nails, irremovable hand accessories, or a disability in both hands were excluded from this study.

### 3.3. Materials

A survey was carried out by face-to-face interviews at the barbecue sites. The participants were required to report their demographic information, instances of handwashing, and their sources of handwashing information.

### 3.4. Procedure

First, information sheets were given to the participants. After obtaining informed consent from them, the participants were asked to rub Glo Germ gel in their hands. After the rater confirmed that both hands were fully covered by the gel, the participants were asked to wash their hands under running water in a way that was typical for them. When the participants were washing their hands, the rater stood far behind them and recorded the time it took them to complete the process. After the participants had washed their hands, a portable, rechargeable black box with 10 watts of UV light was used to assess the residual fluorescent stains on the hands of the participants. The rater then recorded the results.

### 3.5. Research Design and Analysis

International Business Machine (IBM) Statistical Product and Service Solutions (SPSS) for Windows version 24.0 (IBM Corp, 2016) was used for data analysis. The situations in which participants would commonly wash their hands, demographic data, and the duration of handwashing were analyzed by using descriptive statistics of frequency count, mean, and standard deviation. To compare the continuous data between the missed areas and variables in demographic data, independent *t*-test and one-way ANOVA were used. Linear and multiple logistic regressions were used to examine the relationship between variables in demographic data, duration of handwashing, and coverage of missed areas during handwashing. Variables were presented as regression coefficients. The odds ratio was adjusted with corresponding 95% confidence intervals and *p* values. The statistical significance of *p* < 0.05 was taken into account. All statistical tests were two-tailed.

## 4. Results

Eventually, 190 (94 males and 96 females) valid data were collected from the barbeque sites in three main regions of Hong Kong, comprising 47 (25%) participants from Hong Kong Island, 59 (31%) from Kowloon, and 84 (44%) from the New Territories. The ratio of the number of participants in these three geographical areas was similar to the total population distribution of Hong Kong. The characteristics of the samples are shown in Table 1.

### 4.1. Handwashing Survey

**Figure 1:**
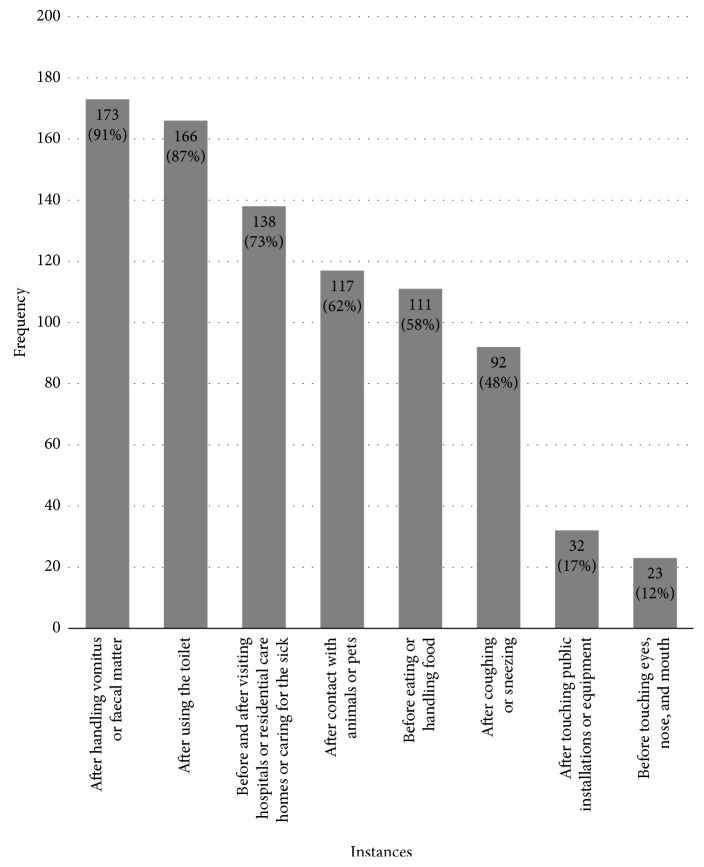
Frequency of 8 handwashing instances collected during the self-reported survey.

The leaflet devised by the CHP advises Hong Kong people to wash their hands in at least 8 different instances. They are as follows: (1) after handling vomitus or faecal matter, (2) after using the toilet, (3) before and after visiting hospitals or residential care homes or caring for the sick, (4) after contact with animals or pets, (5) before eating or handling food, (6) after coughing or sneezing, (7) after touching public installations or equipment, and (8) before touching eyes, nose, and mouth. Among the 190 participants of this study, half (52.6%) washed their hands in 5 of the 8 instances and only 3 participants (1.6%) washed their hands in all 8 instances. The results showed that more than half of the participants washed their hands after handling vomitus or faecal matter (91.1%), after using the toilet (87.4%), before and after visiting a hospital or a residential care home or caring for the sick (72.6%), after having contact with animals or pets (61.6%), and before eating or handling food (58.4%). In contrast, less than half of the participants washed their hands after coughing or sneezing (48.4%), after touching public installations or equipment (16.8%), and before touching their eyes, nose, and mouth (12.1%) (Figure 1).

Using an independent *t*-test and an analysis of variance (ANOVA) to compare the demographic data with handwashing instances, the results showed that the mean handwashing instances of participants with third-level education or above were significantly higher than those of participants who had primary- and secondary-level education only (*p* ≤ 0.001).

### 4.2. Sources of Handwashing Information

Among the 190 participants, 84 (44.2%) received proper hand-hygiene information. Given that this was an open-ended question, participants could list more than one resource. Thus, 106 resources were reported. After grouping and categorising the resources, the result was revealed that the participants obtained information from the media (45%) and from schools (34%); relatively less information was obtained from hospitals (15%) and the workplace (12%). In addition, nearly two-thirds of the youngest age group obtained information from both schools and the media. However, only 31% of the oldest age group received that same information. Moreover, the instances of handwashing of those participants who received informed hand-hygiene information were significantly higher than those of participants who did not (*p* ≤ 0.001). Thus, the mean of the total missed areas was significantly higher in participants who did not receive information about proper hand hygiene than those who received it (*p*=0.004).

### 4.3. Duration of Handwashing

On average, the participants took 36.54 seconds (range = 10–120, SD = 18.57) to wash their hands. The majority of participants (86.8%) washed their hands for longer than 20 seconds, as advocated.

### 4.4. Missed Areas

For each participant, a total of 86 anatomical areas were evaluated. The results showed that the fingertips (48.1%), medial area (30.5%), and back of the hand (28%) were the most commonly missed areas in terms of washing. Using an independent *t*-test and ANOVA to compare the differences between the demographic data and missed areas, we found that unemployed participants had significantly more missed areas than the other participants; the unemployed missed certain areas, including the back of the fingers (*p*=0.014), palms (*p*=0.007), back of the hand (*p*=0.010), and medial areas (*p*=0.003) (Table 2). Full-time university students also neglected the back of the fingers (*p*=0.037). Blue-collar workers had significantly more missed areas, with the medial areas (*p*=0.003) and the lateral areas (*p*=0.005) of the hands being neglected. Moreover, the results showed that participants with third-level education or above (*M* = 10.88, SD = 9.54) had significantly (*p*=0.004) fewer total missed areas than those with primary- and secondary-level education only (*M* = 11.91, SD = 7.94). Among the different age groups, the youngest age group performed significantly better than the older age groups over the front of the fingers (*p*=0.038), the back of the fingers (*p*=0.015), and the lateral side of the hand (*p*=0.012  and  0.019). In terms of the total missed areas, the youngest age group missed significantly fewer areas than the oldest (*p*=0.046).

### 4.5. Multiple Logistic Regression

Multiple logistic regression was used to examine the relationship between the demographic variables, total missed areas, and instances of handwashing. The results showed that age is the only significant predictor of the total missed areas. Those aged 30 and above tended to have 5 more missed areas than those aged 30 or below (*p*=0.001, *B* = 4.933, 95% CI = 2.119–7.747). Considering the variables affecting the total number of instances of hand hygiene, the only significant predictor among the variables was the educational level of the participants. Those with only primary- and secondary-level education tended to have 1 more missed hand-hygiene instance than those whose educational levels were above the third level (*p*=0.001, *B* = 1.003, 95% CI = 0.511–1.495).

## 5. Discussion

### 5.1. Research Implications

Although there is scant empirical evidence on the duration of handwashing for the general public, the key public health agencies around the world, such as CDC, NHS, Public Health Agency of Canada, and Global Handwashing Partnership, adopt the WHO's guideline for the healthcare providers as well as for the community use. In our study, almost 90% of the participants completed their handwashing routine in 20 seconds or more. This contrasts with the studies conducted in the United States by Drankiewicz and Dundes, Shanks and Peteroy-Kelly, and Borchgrevink et al. [4–6].

In 2008, the WHO designed a handwashing leaflet, making reference to Taylor, who indicated that the fingertips, interdigital areas, thumbs, and wrists are the most commonly missed areas in handwashing [26]. Pan et al. also found that the tips of the nails and the fingertips had the largest amount of residual florescent stains left after handwashing among healthcare workers in Taiwan [18]. The commonly missed areas among medical students in the study conducted by Vanyolos et al. was the first metacarpal, the proximal part of the palm (lateral), the distal phalanges, and the nail beds [12]. In healthcare workers in Škodová et al.'s study, the thumbs and fingertips were the most commonly missed areas [27, 28]. In this study, the most frequently missed area was also the fingertips. However, the medial aspect and back of the hand were the second and third most missed areas, respectively. Moreover, the interdigital area and the front and back of the fingers were the least missed areas, which is in contrast to Taylor's study. In terms of gender, there were no significant differences found between male and female participants in our study, which is congruent with Kumar et al.'s study [29]. However, this finding is different from those of Anderson et al., Berry et al., Park et al., Edwards et al., Johnson et al., Ren et al., and Vanyolos et al. [2, 3, 6, 11–14, 30].

In this study, we found that participants with higher educational levels had fewer missed areas, and they performed handwashing on a more regular basis; this is in contrast to the findings of Duggan et al. [15]. However, Curtis et al. in Kenya found that participants who had higher levels of education and literacy had a greater frequency of handwashing [9]. Their study also revealed that media exposure is an important determinant of the frequency of handwashing. In Korea, Park et al. reported that participants who received information about handwashing did wash their hands on a more frequent basis; this finding is in concordance with the finding presented in this study [6]. Furthermore, Curtis et al. also reported that “after defecation,” “before feeding a child,” and “before handling food” were the most common situations in which participants washed their hands [9]. Similar results were also found by Blanton et al. amongst caregivers in Kenyan schools [10]. This was also the case with Ergin et al. in relation to Turkish university students [7]. This study elicited the same results: “after handling vomitus or faecal matter,” “before and after caring for the sick,” and “before eating or handling food” are common situations in which Hong Kong people wash their hands.

In relation to the association between age and hand-hygiene practices, we found that as age increases, handwashing becomes more neglected. However, Burnett highlighted that participants aged 55 years and older had good perceptions of hand hygiene, while the age group with the poorest perceptions was those under 26 years of age. [16] The results of this study may be explained by the intense focus on hand-hygiene education in primary and/or secondary schools after the SARS attack in 2003 but relatively less in the community.

### 5.2. Research Limitations

This study did have its limitations. One of these limitations is the potential unease felt by the participants that evolved by asking for ad hoc handwashing at the venue. Therefore, the researcher did stand far behind the participants, and the participants were not informed that the time spent washing their hands was being recorded. Given the researcher stayed far behind the participants, their compliance with the steps of handwashing could not be evaluated. Besides, although the self-report survey is the fastest way to gather abundant data, this method cannot avoid social-desirability bias in which the participants wanted to “be good,” even though the survey is anonymous. Hence, these two unpreventable conditions may produce potential influences on the results. Furthermore, since most people had barbeque at sunny weekends only, suitable weather and period were quite short which limited the sample size.

## 6. Conclusion

The results provide insights that will be useful for public health and primary-care professionals when reviewing health-promotion strategies for proper hand hygiene. Although the sample size of this study was not large, this study and Pan et al.'s Taiwanese study found that the fingertips are the most commonly neglected areas [18]. Given that Taiwan and Hong Kong are located in the same region, it is worth investigating if similar results are found in other Asian countries. Thus, the reinforcement of fingertip washing is necessary for future handwashing practices. In addition, hand-hygiene education in schools seems to be quite effective; however, hand-hygiene education within the community seems inadequate, particularly for older adults and less well-educated groups.

According to the World Bank in 2018, the population density of Hong Kong increased from 6,587 people per square kilometre in 2007 to 7,040 in 2017, and it ranks as the fourth highest one in the world. People live more closely together than before, and population will increase in the future. Since the outbreak of seasonal influenza and hand-foot-mouth disease has been attacking Hong Kong frequently in recent years, the high population density of Hong Kong may exacerbate the risk of contracting communicable diseases through common contact surfaces such as door knobs, elevator buttons, handrails of escalators, and public transport. In addition to the effects of globalisation, other developed countries or cities with high population density may also be facing similar challenges. Therefore, the public health and primary-care professionals may consider reviewing the protocol for proper hand hygiene and strategy of education and promotion and examine its effectiveness.

## Figures and Tables

**Table 1:** Characteristics of participants who filled out the survey.

	*n* = 190	
	Frequency	%
*Gender*		
Male	94	49.5
Female	96	50.5

*Age*		
18–29	86	45.3
30–39	48	25.3
40–49	24	12.6
50 or above	32	16.8

*Educational level*		
Primary	13	6.8
Secondary	68	35.8
Tertiary or above	109	57.4

*Occupation*		
Unemployed	24	12.6
Full-time students	37	19.5
White collar	72	37.9
Blue collar	36	18.9
Others	21	11.1

**Table 2:** Frequency of missed areas after participants washed their hands.

Missed areas	%
Fingertips	48.1
Medial	30.5
Back of the hand	28
Palm	22.1
Lateral	22
Wrist	18.3
Interdigital	11
Back fingers	5.2
Front fingers	2.5

## Data Availability

All the quantitative data used to support the findings of this study are available from the corresponding author upon request.
